# Visualization and Estimation of 0D to 1D Nanostructure Size by Photoluminescence

**DOI:** 10.3390/nano14241988

**Published:** 2024-12-12

**Authors:** Artūrs Medvids, Artūrs Plūdons, Augustas Vaitkevičius, Saulius Miasojedovas, Patrik Ščajev

**Affiliations:** 1 Insitute of Technical Physics, Riga Technical University, Paula Valdena 3/7, 1048 Riga, Latvia; arturs.pludons@rtu.lv; 2 Institute of Photonics and Nanotechnology, Faculty of Physics, Vilnius University, Saulėtekio Ave. 3, 10257 Vilnius, Lithuania; augustas.vaitkevicius@ff.vu.lt (A.V.); saulius.miasojedovas@ff.vu.lt (S.M.)

**Keywords:** visualization, nanocone, photoluminescence, confinement, recombination

## Abstract

We elaborate a method for determining the 0D–1D nanostructure size by photoluminescence (PL) emission spectrum dependence on the nanostructure dimensions. As observed, the high number of diamond-like carbon nanocones shows a strongly blue-shifted PL spectrum compared to the bulk material, allowing for the calculation of their top dimensions of 2.0 nm. For the second structure model, we used a sharp atomic force microscope (AFM) tip, which showed green emission localized on its top, as determined by confocal microscopy. Using the PL spectrum, the calculation allowed us to determine the tip size of 1.5 nm, which correlated well with the SEM measurements. The time-resolved PL measurements shed light on the recombination process, providing stretched-exponent decay with a *τ*_0_ = 1 ns lifetime, indicating a gradual decrease in exciton lifetime along the height of the cone from the base to the top due to surface and radiative recombination. Therefore, the proposed method provides a simple optical procedure for determining an AFM tip or other nanocone structure sharpness without the need for sample preparation and special expensive equipment.

## 1. Introduction

Nanostructures, such as quantum dots—0D, quantum wires—1D, and quantum wells—2D, are the most prominent objects of research in solid-state physics due to the quantum–dimensional effect. Thanks to this effect, new optoelectronic devices can be created. On the other hand, the tendency to reduce the sizes of artificial light sources enabled the use of such sources in microelectronics, medicine, and biology to study micro-objects such as bacteria and viruses. At the same time, demands have increased to improve the source intensity, the spectrum of radiation, and the uniformity and controllability of its parameters. Spectroscopic micro-objects, such as microbes, molecules, and viruses, require non-traditional research methods, such as an optical probe [1], since the dimensions of such objects are smaller than the wavelength of visible light. Studying micro-objects such as 0D quantum dots requires a light source much smaller than the object. The 2023 Nobel Prize award in Chemistry to Mango J. Bawendi, Louis E. Bruce, and Oleksiy I. Yekimov “for the discovery and synthesis of quantum dots” confirms that the quantum confinement effect occupies the dominant place in the solid-state physics [2,3,4]. Opto-electronic devices and technologies based on the quantum confinement effect in quantum dots or quantum wires are the main tasks for scientific development [5]. We have shown the possibility of forming quantum cones on the surface of Si, Ge, GaAs, CdTe, and diamond-like carbon (DLC) [6,7,8,9,10,11,12]. A quantum cone consists of many quantum dots whose diameters gradually increase from the top to the base of the cone, leading to a dispersive radiated spectrum. Therefore, the diameter and energy change of the excitons in the cone [13] leads to their lifetime dependence on the vertical position.

**Figure 1:**
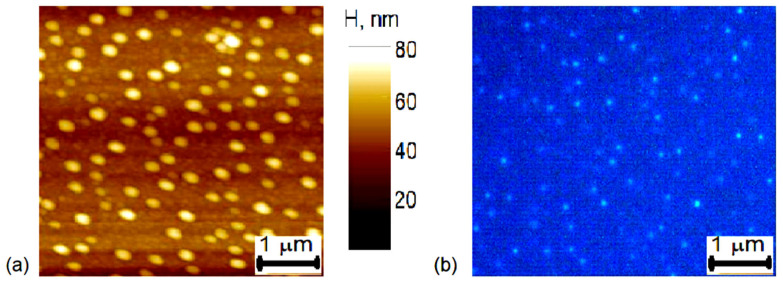
AFM (**a**) and the fluorescent microscope (**b**) images of a DLC sample at UV excitation for the same surface.

**Figure 2:**
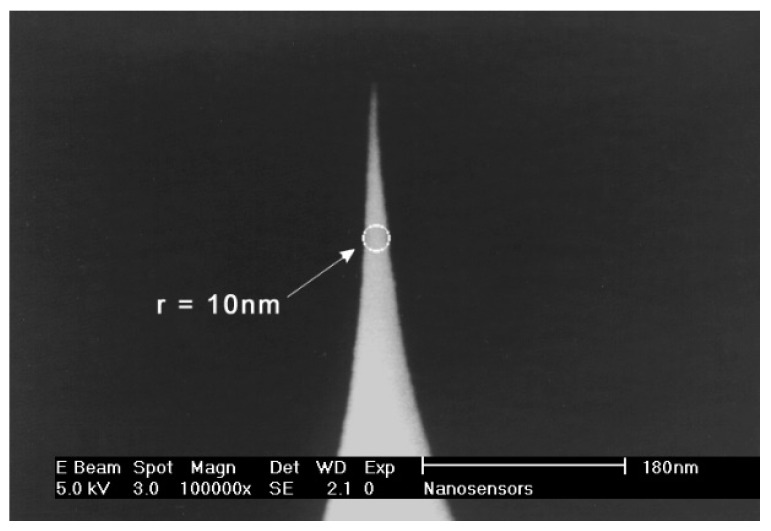
SEM image of AFM Si probe for surface research, manufactured by NANOSENSORS ^TM^.

**Figure 3:**
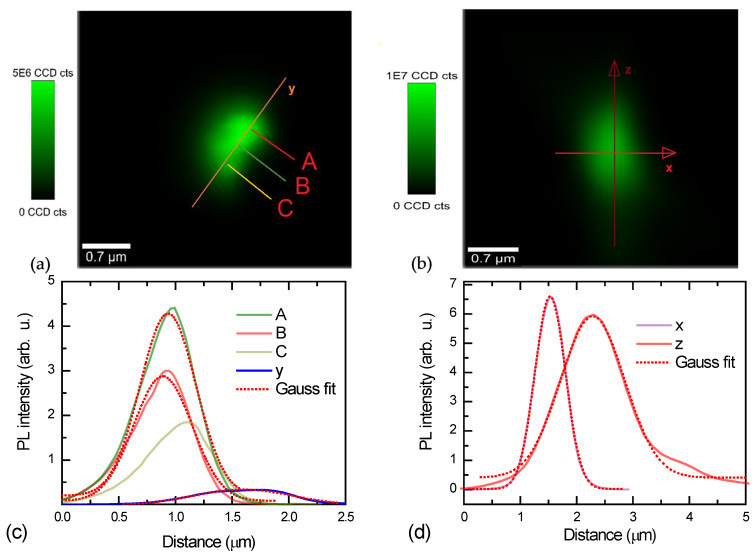
Confocal optical microscope image of Si tip No. 1 photoluminescence in the horizontal (**a**) and vertical (**b**) cross-sections at 405 nm excitation with 0.25 mW power. Cross-section intensities in horizontal (**c**) and vertical (**d**) scans for indicated directions; in A, B directions, Gauss fit provides FWHM 640 nm and 570 nm; in x, z directions, FWHM is 640 nm and 1430 nm, respectively.

The application of nanocones is very wide in science and industry; for example, in an atomic force microscope (AFM), they are used in the probe tip. The aim of this study is to visualize and estimate the diameter of an invisible nanocone tip, as shown in Figure 1, Figure 2 and Figure 3. This is an important task for the user and the manufacturer, for example, when measuring the diameter of the AFM tip, as shown in Figure 2, when making it for AFM and manipulating it. In situ studies of AFM probes by SEM are impossible because it is necessary to use high vacuum and sensitive electron emitters and detectors.

## 2. Materials and Methods

The DLC films with nanocones were formed by magnetron sputtering C on the Si substrate and subsequent thermal annealing at *T* = 1060 °C in the N atmosphere [12]. The thickness of the DLC layers was 400 nm. The annealing of such samples leads to the formation of 80 nm—high DLC nanocones on the DLC film, according to the Stranski–Krastanow model. The nanocones are observed by AFM (see Figure 1a) and visualized by a fluorescent microscope (Olympus BX51 Fluorescence Microscope (Tokyo, Japan), 100×, NA = 1.4 objective, see Figure 1b) at UV excitation. The light points are shown in Figure 1. Estimation of the diameter of the nanocone tops on the surface of sample 3A, using the formula *E_g_* = *E_g0_ +* 1.42*h*^2^/(4*μd*^2^) from paper [14], gives *d* = 2.0 nm [12]. In this formula, *E_g_* = 3.3 eV is the blue-shifted bandgap due to the quantum confinement; *E_g0_* is the DLC material bandgap of 2.8 eV, and *μ* = 0.43 *m*_0_ is its reduced effective mass.

In this work, we selected a single AFM tip to distinguish its properties from the substrate. The AFM probe was produced by Nanosensors, a model of SuperSharpSilicon^TM^ SSS-NCL (NANOSENSORS™, Neuchatel, Switzerland) [15]. It features doped silicon to dissipate the static charge, half cone angle at 200 nm from apex: <0°, and a typical top radius of less than 2 nm. Three probe pieces were analyzed: No. 1, No. 2 and No. 3.

The confocal photoluminescence (PL) measurements of the AFM probe were performed using the WITec Alpha 300S microscope (Ulm, Germany) coupled with a UTS-300 spectrophotometer (Andor, Belfast, UK) equipped with an air-cooled CCD camera. The excitation light was blocked with a long pass edge filter BLP01-405r (IDEX Health & Science, New York, NY, USA). Excitation was performed by a 405 nm waveguide Alphalas laser (Alphalas, Goettingen, Germany). A 100× microscope objective with NA = 0.9 was used for both the excitation and emitted PL light collection.

The time-resolved PL measurements were performed by a Hamamatsu streak camera C10627 (Hamamatsu, Japan) connected to an Acton monochromator with 300 cm focal length (Princeton Instruiments, Acton, MA, USA). Data were collected in a photon-counting regime in a 20 ns time window. Excitation was performed by 350 nm and 200 fs duration laser pulses generated by ORPHEUS parametric generator pumped by PHAROS laser (Light Conversion, Vilniaus, Lithuania) at a 10-kHz frequency. The excitation intensity was controlled by a continuous neutral density filter. The scattered excitation light was blocked by a long-pass filter.

## 3. Results and Discussion

**Figure 4:**
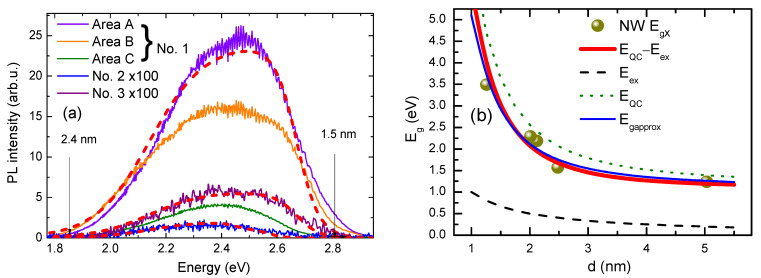
(**a**) PL spectra of Si tip No. 1 in A, B, and C areas exited by UV laser at λ_ex_ = 405 nm and 0.25 mW power; PL spectra of tips No. 2, No. 3 magnified by 100 times. The dashed line shows a fit by Equation (2). (**b**) Nanowire bandgap dependence on its diameter [19] and fitted contributions of quantum confinement and exciton binding energies. Red curve shows a total fit by Equation (1).

The sharp SSS-NCL AFM probe is shown in Figure 2 by SEM and in Figure 3 by confocal photoluminescence microscopy. The confocal image shows green emission localized at the AFM probe top with few emitting points. Due to the microscope resolution of ~500 nm, the image is blurred and cannot be used to determine the AFM tip dimensions. The confocal vertical cross-section confirms that the PL emission is observed only from the tip’s top. The confocal microscopy PL spectra of the studied tip are provided in Figure 4a. The nanotip PL intensity cross sections observed with a confocal microscope are provided in Figure 3c,d. The full width at half maximum (FWHM) of the x cross-section is 640 nm for the confocal microscope, which exceeds the theoretical one of 0.51 × *λ_PL_*/NA = 300 nm, calculated for *λ_PL_* = 500 nm at the peak PL emission wavelength and NA = 0.9. This is because the microscope employs a waveguide with a large aperture to collect light. The luminescing nanotip size of a few nm is much smaller; thus, the diffraction limits the image size. In the y direction, the image is prolonged into 1200 nm (which is close to the vertical cross-section resolution of 1430 nm—z direction), which can be plausibly caused by the emitted light waveguiding to the bottom of the nanotip and scattering there from surface defects, thus leading to the peak red-shifts (points B, C) as the shorter wavelengths are more absorbed in the silicon tip. Therefore, point A, at the top, with the most blue-shifted spectrum, was selected for the tip diameter analysis. For practical use, simply green photoluminescence can be detected by using a built-in blue laser (focused on the tip) and a camera in the AFM microscope providing the in situ measurements, which is much simpler than SEM analysis.

In the case when it is necessary to estimate the diameter of the nanocone at any height of the tip, the quantum confinement ∆*E_G_* = *E_QC_* formula from paper [16] for nanowires can be used. The bandgap energy *E_g_* in the direction of the height *z* of the cone gradually decreases from the top of the cone to its base as a function of the diameter of a nanowire [16]. Including the approximated exciton binding energy for Si nanowires from [17,18] into the formula from paper [16], we obtain the following:

(1) $$\begin{array}{l} E_{g}\left(z\right)=E_{g0}+E_{QC}\left(z\right)-E_{ex}\left(z\right),\\ E_{QC}=\frac{2\hbar^{2}\zeta^{2}}{m^{*}d^{2}\left(z\right)};E_{ex}=\frac{2e^{2}}{\pi \varepsilon_{0}\varepsilon_{s}d\left(z\right)}, \end{array}$$

where *E_g0_* = 1.16 eV is the Si bandgap, and *ε_s_* = 11.9 is the Si dielectric constant. *E_QC_* and *E_ex_* are the quantum confinement and exciton binding energies, respectively, depending on the nanowire diameter *d*. Here, the inverse effective mass is described as 1/m* = 1/m_e_* + 1/m_h_* (m_e_* = 0.26 *m*_0_ and m_h_* = 0.47 *m*_0_ are silicon electron and hole effective-masses, respectively; m* = 0.167 *m*_0_). For quantum wires, *ζ* = 2.4048 [16]. Equation (1) correctly fits the experimental change in the Si nanowire bandgap data from [19] and can be approximated by a simplified relation of excitonic bandgap *E_gapprox_*(*d*) = 1.1 eV + 4 × 10^−18^ eVnm^2^ × 1/*d*^2^, which is also shown in Figure 4b. In our case, the diameter of the nanowires is a function of the height *d*(*z*). Thus, it is a semiconductor structure with a graded band gap. Calculation of the cone tip size using Equation (1) in the highest point A and the largest *E_g_* emission from the PL blue cutoff at *E_gmax_* = 2.8 eV in Figure 4a gives the top point A diameter of *d* = 1.5 nm. The peak PL emission at 2.45 eV provides the diameter of *d* = 1.72 nm, where the emission is the most efficient. The tips No. 2 and No. 3 show fitted top point diameters of *d* = 1.9 nm and *d* = 1.6 nm, respectively. Much smaller PL intensity in the latter tips may indicate larger surface defect density. The diameter roughly agrees with the sharp AFM tip dimensions observed with SEM (Figure 2). Nevertheless, SEM measurements show larger dimensions due to the natural oxide covering the nanotip. At room temperature, oxidized nanowire has external dimensions of ~3 nm, as found by calculation [20], and the internal Si nanowire diameter is minimally about 1.2 nm, which considerably agrees with our results.

To explain the broad PL spectra observed in Figure 4, the following equation can be used:

(2) $$I_{PL}\left(E\right)=A\times \int_{0}^{\infty }\frac{{\left(E-E_{g}\left(d\left(z\right)\right)+E_{TO}\right)}^{2}}{1+\exp\left(\left(E-E_{g}\left(d\left(z\right)\right)\right)/kT\right)}\frac{dz}{\tau_{rad}\left(d\left(z\right)\right)}.$$

Here, *A* is an arbitrary fitting amplitude; *T* is the temperature. Integration is stopped at *z* = *d_min_*, corresponding to *E_gmax_* as the maximum excitonic bandgap at the cone top. Here, *E_TO_* = 58 meV is the transverse optical phonon energy of Si, which dominates the PL emission [21]; in nanowires, optical phonon spectra and energies do not change significantly [22]. The exciton emission has a strongly diameter-dependent lifetime, which is described by an approximate relation [23]: *τ_rad_*(*d*) = 1/(0.05 × (1 + cos(10^10^nm^−1^ × *d*(*z*))) × exp(−0.5 × 10^10^nm^−1^ × *d*(*z*)) + 1.2 × 10^−8^) × 10^−11^ s. The lifetime reduces with *d* many orders of magnitude due to the exciton confinement. That was already observed on variable diameters of nanowires where the lifetime was reduced from milliseconds to nanoseconds [24]. The first term in the integral of Equation (2) consists of the thermal broadening function [21] smearing cutoff of the spectrum.

**Figure 5:**
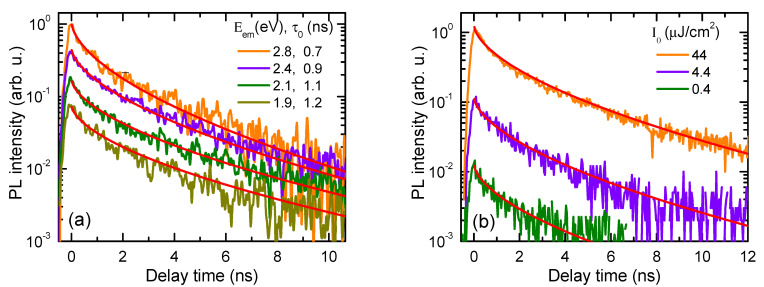
(**a**) Kinetics of PL for selected emission energies E_em_ for tip No. 1. Red-colored curves show stretched exponent fit by equation *I*_*PL*_ = *I_PL_*_0_ exp (−(*t*/*τ*_0_)*β*), where *β* = 0.58, and τ_0_ is the initial lifetime of excitons at *I*_0_ = 44 μJ/cm^2^ excitation; curves are vertically shifted for clarity. (**b**) Excitation-dependent PL kinetics averaged over emission spectrum; solid lines show that stretched exponent fits with *β* = 0.58 and *τ*_0_ = 1.0 ns and relative amplitudes *I_PL_*_0_ = 1:0.1:0.01.

The kinetics of PL are provided in Figure 5. They possess nonlinear behavior, which was fitted by a stretched exponent function with an average lifetime of *τ_o_* = 1.0 ns. The decays at different emission wavelengths were rather similar, with slight variation in the initial decay time in the 0.7–1.2 ± 0.1 ns range. The decays were also excitation-independent, indicating that nonlinear recombination processes did not impact the exciton transport, as excitation was relatively low. The dispersion parameter *β* = 0.58 value is rather close to the observed in DLC nanocones, with *β* = 0.55. Therefore, the nonexponentiality of the decay could be explained by the drift of the excitons from the cone top to the base of the cone due to the built-in electric field caused by the bandgap gradient as in [25]. However, the DLC cones showed very strong decay time dependence on the emission wavelength [25]; therefore, in the case of the Si AFM tip, another mechanism could come into play. Due to the small diameter of the tip and small cone angle, surface recombination can have a considerable impact. Using the surface recombination lifetime equation for a cylinder with a *d* diameter, the surface lifetime can be described as *τ_S_* = 0.282*d/S* [26]. According to the determined 1 ns initial lifetime, we can evaluate the surface recombination velocity of *S* = 1.1 cm/s (in tips No. 2 and No. 3, the PL signal intensities are 1700 and 400 times smaller, plausibly indicating much larger *S* values of 1700 cm/s and 400 cm/s, respectively). Low *S* values (<1 cm/s) are typical for high-quality SiO_x_ passivation [27]. At shorter wavelengths, emission appears from the smaller diameter part of the needle. The emission decay at 1.9 eV is 1.7 times slower than at 2.8 eV (Figure 5), which correlates with the larger diameter of *d* = 2.4 nm on the base vs. 1.5 nm on the tip, therefore confirming the weak *τ_S_ ~ τ_nonrad_* dependence on *d*. The radiative lifetime of excitons has a much steeper dependence, as mentioned before. The radiative efficiency (Φ = 1/(1 + *τ_rad_/τ_nonrad_*) *= τ*_0_*/τ_rad_*, *τ_nonrad_*, and *τ_rad_* are the nonradiative and radiative lifetimes, respectively) of the tip was determined to be around 15%, confirming this hypothesis and 1*/τ*_0_ *=* 1*/τ_rad_ + 1/τ_nonrad_ ~*1*/τ_nonrad_*. From this relation, we can evaluate the average radiative lifetime of ~ 6 ns, which is similar to ~12 ns in highly luminescent Si nanocrystals with ~2 nm size [28]. Notably, the decay tail lifetime in Figure 5 reaches 4 ns, being close to the radiative lifetime, which can indicate saturation of the surface defects.

**Figure 6:**
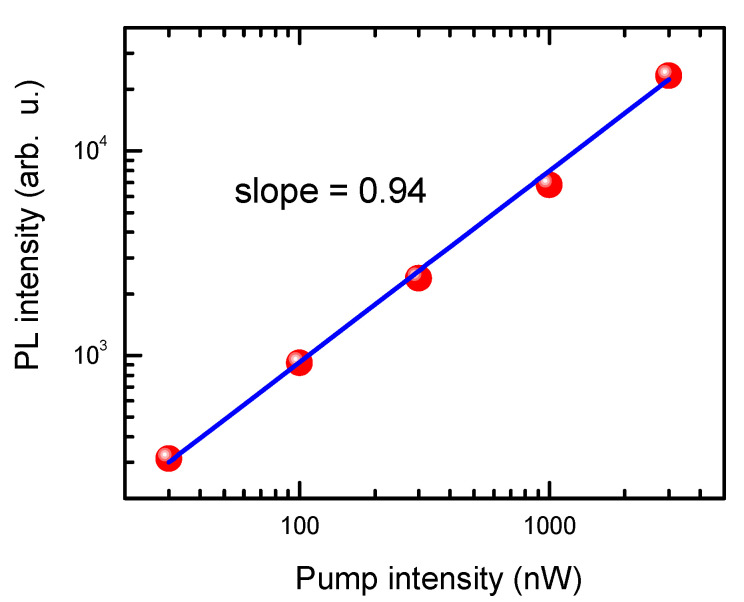
Peak PL intensity as a function of pump excitation intensity (tip No. 1).

The PL linear slope on excitation (Figure 6) is possible as the silicon cone is n-type-doped and PL is measured at relatively low excitations; thus, *PL ~ N_exc_ ~ n_dop_ × N_ehp_* [29], where *N_exc_* and *N_ehp_* are the densities of emitting excitons and generated electron-hole pairs, respectively. Excitons have high binding energy on the top (Figure 4b); thus, they are the dominant species in PL emission. According to the resistivity of the AFM probe, 0.01–0.025 Ω·cm [15], by using calculations from [30], we determine a high electron density of *n_dop_* = ~10^19^ cm^−3^ in the sample.

## 4. Conclusions

We propose a new method for a 0D–1D nanostructure visualization and estimation of its top diameter. For example, AFM tip dimensions are studied when making it for AFM and manipulating it to check its sharpness. This method is based on the PL emission spectrum of the sharp Si nanoconic AFM probe, inducing exciton localization on its top. The wideband green emission of a super-sharp AFM probe verifies its top dimension of 1.5 nm. Complimentary time-resolved PL measurements provided stretched exponent decay related to the emitting exciton species decay due to their radiative and surface recombination in the AFM tip. The developed method is also well-suited for determining the sharpness of the semiconductor nanocone arrays, e.g., DLC nanocones.

## Data Availability

The data that support the findings of this study are available upon reasonable request from the authors.
